# Particle Size and Rheology of Silica Particle Networks at the Air–Water Interface

**DOI:** 10.3390/nano13142114

**Published:** 2023-07-20

**Authors:** Siddharth Thakur, Sepideh Razavi

**Affiliations:** School of Sustainable Chemical, Biological and Materials Engineering, University of Oklahoma, Norman, OK 73019, USA

**Keywords:** silica nanoparticles, particle-laden interfaces, interfacial rheology, capillary interactions

## Abstract

Silica nanoparticles find utility in different roles within the commercial domain. They are either employed in bulk within pharmaceutical formulations or at interfaces in anti-coalescing agents. Thus, studying the particle attributes contributing to the characteristics of silica particle-laden interfaces is of interest. The present work highlights the impact of particle size (i.e., 250 nm vs. 1000 nm) on the rheological properties of interfacial networks formed by hydrophobically modified silica nanoparticles at the air–water interface. The particle surface properties were examined using mobility measurements, Langmuir trough studies, and interfacial rheology techniques. Optical microscopy imaging along with Langmuir trough studies revealed the microstructure associated with various surface pressures and corresponding surface coverages (ϕ). The 1000 nm silica particle networks gave rise to a higher surface pressure at the same coverage compared to 250 nm particles on account of the stronger attractive capillary interactions. Interfacial rheological characterization revealed that networks with 1000 nm particles possess higher surface modulus and yield stress in comparison to the network obtained with 250 nm particles at the same surface pressure. These findings highlight the effect of particle size on the rheological characteristics of particle-laden interfaces, which is of importance in determining the stability and flow response of formulations comprising particle-stabilized emulsions and foams.

## 1. Introduction

Colloidal systems at interfaces have long attracted the attention of researchers as their assembly can be structured towards designing complex architectures for a myriad of applications [1,2]. In this regard, different works in the literature have focused on the utility of decorated interfaces in sensing, catalysis, and stabilization of complex formulations such as bijels [3,4,5,6]. Particle-laden interfaces, in particular, are critical to the functioning of products, such as paints, pharmaceutical formulations, and emulsions and foams [7]. Pickering emulsion/foam is a well-known representative of particle-stabilized systems [8], in which particles adsorb onto the fluid–fluid interface, irreversibly, owing to their large desorption energy, and they stabilize the droplets/bubbles of the dispersed fluid against coarsening [9]. Various particle attributes, such as wettability, roughness, size distribution, porosity, and shape anisotropy, are shown to affect the resulting microstructure of the particle-laden interface, which determines the suitability of the particles towards different applications [10,11,12,13,14]. Silica particles are studied extensively in this research field owing to their versatile nature [15]. The surfaces of these particles can be tuned from hydrophilic to hydrophobic, and their response can be altered as a function of pH; hence, silica particles are found in a range of applications, from anti-fogging formulations to cement binders [16,17,18].

Understanding the parameters governing the assembly of particles at interfaces is vital towards tailoring the physico-chemical characteristics of networks in order to control the stability of multiphase fluidic systems, such as emulsions and foams [14,19,20,21,22]. Furthermore, in various applications involving multiphase fluids, such as enhanced oil recovery and bio-film degradation [23,24,25,26,27,28], the interface is present in non-equilibrium conditions and is subjected to flows causing shear and compressional stresses [29]. Hence, analyzing the effect of different particle attributes on the microstructure of the network formed at interfaces and its associated rheological response, under the operating conditions of interest, will be beneficial to the design of formulations with a desirable set of properties. The interfacial rheology of particle-laden interfaces provides an insight into the dynamic mechanical characteristics that determine the stability of the particle monolayer under various conditions, such as shear (varying the shape of the interface while keeping its area constant) and dilatation (varying the interfacial area while retaining its shape) [29,30].

Different factors, including the particle surface coverage [31,32], wettability [33], polydispersity [34], shape [35,36,37], and surface anisotropy [38,39,40], are shown to impact the mechanical properties of the interface. For instance, Christopher et al. [31] studied the effect of particle surface coverage (ϕ) on the interfacial microstructure and its related rheological properties using sulfate polystyrene particles (diameter~3 µm) at the air–water interface (particle 3-phase contact angle, θ~117°). Using oscillatory shear strain sweeps, it was found that reducing the surface coverage from 86% to 56% resulted in a decrease of ~2 orders of magnitude in viscoelastic storage modulus. Image analysis revealed that at high-surface-concentration regimes, lower strains led to the appearance of hexagonal domains (structure factor curves indicated hexagonal order), and increasing the strain resulted in the loss of long-range hexagonal order. As the surface concentration reduced, an increase in the strain value led to the occurrence of more defects in packing (structure factor curves indicated transition to isotropic structure).

Particle wettability not only affects the 3-phase contact angle of the particles at the interface and the resulting binding energy to the interface but also influences the interparticle interactions, which are a determining factor in the mechanical characteristics of the particle network. Binks et al. [33] utilized 20 nm silica nanoparticles at various degrees of hydrophobicity, where particle wettability was altered by changing the particle surface silanol (SiOH) content in a range of 20–100%. Under the applied compressions, more hydrophobic particles were observed to form compact domains, in comparison to hydrophilic particles, which formed cloud-like aggregates. In addition, the particle concentration required to cover the entire surface was the least for particles with intermediate hydrophobicity of (i.e., 34% SiOH), which displayed the highest surface pressure for equivalent area. This was attributed to the fact that these particles are trapped at the interface with θ~90° and possess relatively stronger attractive interparticle interactions in comparison to particles of other wettabilities. Binks et al. [41] also reported in an earlier study that for fumed silica nanoparticles (20 nm) with different hydrophobicities, increasing the silane grafting density on the particle surface enhanced the capillary interaction between the particles at the air–water interface, leading to the formation of interfacial networks with larger compression and shear elastic moduli. A higher degree of heterogeneity leads to long-range attractions due to the distortion of the contact line. Razavi et al. [42] studied the response of interfacial networks, formed by 1 µm functionalized silica particles, to large compressional strains and found that particles with a higher degree of hydrophobicity (θ~97°) formed a solid-like compact network that collapsed via buckling, whereas the interfacial microstructure that resulted from more hydrophilic particles (θ~57°) behaved in a liquid-like manner, in which particles were expelled from the interface under compression.

Switching from an air–water interface to an oil–water system is also shown to alter the microstructure of the particle network formed at the interface and has been attributed to Columbic repulsive interactions through the oil phase [43,44]. Horozov et al. [45] studied the behavior of hydrophobically modified silica particles (1 µm and 3 µm) at the oil–water interface. Different degrees of hydrophobicity were imparted on the particles via a silanization reaction to obtain contact angles in a range of 70°–150°. As the particle contact angle increased (i.e., more hydrophobic particles), a more ordered monolayer was obtained due to enhanced Coulombic repulsion through the octane oil phase. When comparing the two particle sizes of similar contact angles, for 1 µm particles (θ~150°), the hexagonal ordering resulted in an interparticle distance of ~6 particle diameters, while for 3 µm particles (θ~148°), the spacing was ~3 particle diameters. Therefore, particle size could impact the strength of interparticle interactions and, thus, affect the mechanical properties of the network.

In addition to the studies available on relatively monodisperse particles, investigations have also been performed on systems involving mixtures of particles of different sizes to provide insight regarding particle assembly and corresponding phase transitions. Ata et al. utilized soda lime particles of sizes 64 µm, 96 µm, and 150 µm that were surface-modified to attain a contact angle of θ~64° at the air–water interface [34]. It was discovered that the bimodal network layer consisting of a higher fraction of large particles possessed more voids, presented a random packing, and had a higher compressibility, compared to those obtained from particle samples with a unimodal size distribution. Weiss et al. [46] studied the impact of having a binary particle system on the microstructure of the resulting particle layer at the interface and its response to compressional stresses using latex particles of diameters 202 nm and 1063 nm and particle number ratios (*N*_small_/*N*_large_) of 2, 6, and 9. It was reported that surface pressure isotherms formed by different number ratio combinations resembled the isotherm obtained for a monodisperse distribution of the larger particles. This finding suggested that the interfacial properties were dictated by the larger particles, which formed a dense packing, along with the smaller particles that occupied the interstitial space.

While reports are available in the literature on the impacts of various particle attributes on the resulting interfacial microstructure at the air–water interface [31,32,33,34,41,42,47,48,49,50,51], understanding how the particle characteristics affect the resulting interparticle interactions and how to exploit these attributes in engineering the rheological properties of such interfacial particle network is an active area of research [52,53]. Moreover, the potential environmental concerns associated with some nanoparticles are driving a shift towards the use of larger particles in applications [54]. The present study is focused on the effect of particle size on the microstructure of the interfacial networks formed by 250 nm and 1000 nm silica particles at the air–water interface and their subsequent interfacial mechanics. The interfacial particle network formation is examined via optical microscopy. The rheological characteristic of the resulting network is investigated under applied deformations in both dilatation and shear modes. An analysis of the inter-particle interactions at play is carried out in order to link the attributes of the particles used in this study to the respective networks formed at the air–water interface.

## 2. Materials and Methods

### 2.1. Materials

Silica particles with nominal diameter of 250 nm and 1000 nm were purchased from Fiber Optic Center Inc. The size of particles is measured to be 267 ± 17 nm and 987 ± 22 nm, respectively, using a scanning electron microscope (Thermo Quattro S field-emission SEM, Waltham, MA, USA). It may be noted that these measured values of particle sizes are used throughout the work for the calculation of other particle attributes. Untreated silica particles are hydrophilic in nature due to the presence of surface silanol groups and their dissociation in aqueous solutions [55]. In order to impart hydrophobicity to their surface and enhance their interfacial binding, particles underwent silanization reaction [56] in presence of dichlorodimethylsilane (DCDMS, Acros Organics, NJ, USA) following a procedure reported in the literature [45]. Briefly, 5 gr of silica particles was dried overnight in an oven at 60 °C. The particles were then mixed with 40 mL cyclohexane (Fisher Scientific, Hampton, NH, USA) and the mixture was sonicated for 30 min. Next, 0.51 gr of DCDMS was added to the mixture to yield a concentration of 0.1 M in cyclohexane. The mixture was subsequently sonicated for 30 min. Thereafter, the suspension was centrifuged (Sorvall Legend X1R, Thermo Scientific, Waltham, MA, USA) at 5000 RPM (2739× *g*) for 5 min. The particles underwent subsequent washing steps using cyclohexane, chloroform (Fisher Scientific), and anhydrous ethanol (Fisher Scientific), respectively, by redispersing them in the solvent followed by sonication and centrifugation for 5 min each. Lastly, the treated particles were separated and dried overnight in a vacuum oven at 60 °C. The glassware utilized was thoroughly cleaned using 0.1 N KOH (Fisher Scientific) solution. Ultrapure water (Millipore, MA, USA) with a resistivity of 18.2 MΩ.cm was used throughout the study.

### 2.2. Particle Surface Characterization

Following the work of Horozov et al. [45], effect of silane treatment on the resulting surface wettability of particles was estimated by measuring the contact angle of water droplets on base-cleaned glass substrate that went through the same treatment process along with the particle samples. A water droplet (~5 µL) was deposited on the surface of the substrates, and the 3-phase contact angle (θ) was extracted from the droplet shape using a tensiometer (Biolin Scientific, Gothenburg, Sweden).

Hydrodynamic size of the particles was obtained using dynamic light scattering (DLS) instrument (Nanobrook Omni, Brookhaven Instruments, Holtsville, NY, USA). Mobility (μ) measurements were also carried out to track the change in the particle surface charge resulting from the silane treatment. Aqueous dispersions (pH 5.9 ± 0.2, as reported by DLS) of the particles (0.005 wt%) were prepared and sonicated for 20 min followed by the hydrodynamic size and mobility measurements. It may be noted that these measurements in water were carried out after the particles underwent a phase inversion process from ethanol. The particles were initially dispersed in ethanol, centrifuged at 7000 rpm for 6 min, and redispersed in water (the centrifugation/redispersion procedure was repeated twice). Zeta potential measurements on treated particles provided mobility (μ) values, which, along with the conductance values (χ), were used to calculate parameters, such as charge density (σ) and Debye length ($\kappa^{-1}$). Specifics on the calculations of these parameters are provided in the Supporting Information. The Debye length ($\kappa^{-1}$) of the solutions, measured from the conductivity values reported by the DLS, was measured to be $\kappa^{-1}\;$~130 ± 14 nm. Henry’s function was utilized to determine the zeta potential of particles based on the measured mobility values. Mobility measurements were also carried out on particles dispersed in ethanol; the calculated zeta potential corresponding to these measurements is provided in the Supporting Information (see Figure S1).

### 2.3. Microstructure of Particle Networks at the Air–Water Interface

Experimental studies on the microstructure of the interfacial particle layers were carried out using a Langmuir trough (Biolin Scientific, Gothenburg, Sweden) with a working area of 150 cm^2^. The interfacial area (A) was varied in a range of 25–150 cm^2^ using Teflon barriers, while the resulting surface pressure (Π) was measured via the Wilhelmy plate oriented parallel to the barriers and attached to a pressure sensor. The surface pressure is defined as the difference between the air–water surface tension ($\gamma_{0}$) and the effective surface tension in presence of particles ($\gamma_{eff}$). Before each measurement, the trough and barriers were cleaned thoroughly with ethanol and DI water. In order to ensure contamination-free analysis, the trough was filled with DI water, compressed to a working area of 25 cm^2^, and the interface was aspirated repeatedly until the effective surface pressure rise at the compressed state remained below 0.3 mN/m.

To prepare the particle-laden interfaces, silica particles were dispersed in ethanol at a concentration of 37.5 mg/mL and sonicated for 20 min before deposition at the interface. Dropwise deposition of the resultant dispersion at the air–water interface was carried out using a Hamilton syringe. For 250 nm particles, 100 μL of the dispersion was gradually deposited, whereas for 1000 nm particles, 300 μL was deposited. The amounts of particle deposited were chosen such that similar range of surface pressures (0–40 mN/m) could be attained by the compression of the network on a Langmuir trough. After a waiting period of 30 mins, provided for ethanol evaporation, the barriers were compressed at a rate of 10 mm/min (15 cm^2^/min) to obtain the pressure–area isotherms for each particle network. Reducing the compression rate to smaller values did not change the resulting surface pressure isotherms. To analyze the strength and compressibility of the particle network, static compressional modulus ($\kappa_{0}^{s}$) of the surface layer was determined from the pressure–area isotherms at constant temperature according to Equation (1) [12],

(1) $$\kappa_{0}^{s}=-A{\left(\frac{\partial \Pi }{\partial A}\right)}_{T}$$

The generated surface pressure isotherms could be linked to the particle network formation at the interface, as a function of particle surface coverage (ϕ), using images captured by an Olympus IX-73 inverted optical microscope and an sCMOS camera (Hamamatsu ORCA Flash 4.0 LT+, Tokyo, Japan). The optical micrographs were captured with a 10X objective (N.A. 0.25, W.D. 10.6 mm) during the compression experiments. The surface pressure isotherm data were then fed to a MATLAB algorithm developed to determine the inflection point of the isotherm. In the available literature on particle-laden interfaces, the inflection point (IP) has been linked to the transition of the particle layer from 2-dimensions to 3-dimentions, in response to the applied compression, followed by its collapse [42,57,58,59]. Assuming a random closed-packing arrangement for particles at the inflection point of the isotherm ($\phi_{IP}$ = 0.89) [60], the particle packing fraction at the interface can be determined for different surface areas along the isotherm, as presented in this work.

### 2.4. Interfacial Shear Rheology

The shear rheology experiments were conducted via a DHR2 stress-controlled rheometer (TA Instruments, New Castle, DE, USA) using double wall ring (DWR) geometry. The setup was integrated with a Langmuir Ribbon trough (working area 150 cm^2^) following a procedure developed by Vermant and coworkers [61]. The same procedure used for depositing the particles on the Langmuir trough was employed to prepare the particle monolayers for interfacial shear rheology measurements. Next, the interfacial layer was compressed to achieve surface pressure values of 10, 20, 30, and 40 mN/m. Oscillatory measurements were carried out at these surface pressures to analyze the rheological characteristics of the particle network at the air–water interface. Amplitude sweeps were conducted at 1 Hz to identify the linear viscoelastic (LVE) regime, and the frequency sweeps were performed at 0.01% strain (within the LVE region). The operating window for the oscillatory measurements that leads to valid reproducible results was calculated following the work of Vermant and coworkers [62] and was color coded in the reported figures. In brief, the data falling in the green range represent an acceptable data set, the data falling in the yellow region represent probably acceptable data, and the rest, falling in the red region, needs to be handled with care. Further information regarding determination of the limits is provided in the Supporting Information.

### 2.5. Interfacial Dilational Rheology

To study the dilational rheological characteristics of the particle monolayer at the air–water interface, a tensiometer setup connected to a piezo-actuator device (OneAttension, Biolin Scientific, Gothenburg, Sweden) was utilized. The interfacial particle network was formed at a droplet surface following a procedure described in the literature [63]. Briefly, a water droplet (13 ± 1 μL) was generated at the tip of a 12-gauge needle (outer diameter~27 mm) forming a pendant drop. Next, a secondary droplet (3 μL) of the particle dispersion in ethanol (37.5 mg/mL) was deposited at the surface of the pendant drop. The particle-laden pendant drop was, thereafter, injected with water to reach a final drop volume of 44 ± 2 μL and left undisturbed for 30 min for the ethanol to evaporate. As the drop volume reduced due to evaporation of ethanol, water was reinjected into the drop to keep the volume at ~45 μL. Next, the drop shape was analyzed as a function of time, as the digitized pendant shape was fit according to the Young–Laplace equation, with the aid of OneAttension software (https://www.biolinscientific.com/attension/optical-tensiometers/theta-pulsating-drop#software, accessed on 10 July 2023). To carry out tensiometry measurements, the droplet volume was reduced stepwise, which translated into a reduction in the interfacial area available to the particles and imparted a change in the surface pressure. At various surface pressures, in a range of 1–35 mN/m, the particle-laden drop was subjected to small-amplitude oscillatory perturbations in volume, corresponding to oscillations in the interfacial area with a strain amplitude in the range of 0.3 ± 0.1% to 1.2 ± 0.3%. The droplet was oscillated at frequencies of 0.05, 0.1, 0.5, and 1 Hz, at each surface pressure. The change in the pressure of interfacial layer in response to the applied oscillations and its phase shift (δ) with regard to the perturbations in the area were used to calculate the complex surface dilatational moduli ($\kappa^{s*}$) of the particle network, from which the elastic ($\kappa^{s\prime }$) and viscous ($\kappa^{s\prime \prime }$) contributions are derived. After determining the surface dilatational moduli at one surface pressure, the drop was compressed in a stepwise fashion, at pressure increments of 2–3 mN/m and at a fixed rate of 0.5 μL/s, to reach the next surface pressure value of interest at which the oscillatory measurements are repeated. After every compression, a wait period was employed until no significant relaxation in the surface pressure was observed. It may be noted that reliable results are obtained when the analysis is performed on particle-laden droplets characterized by Worthington number, as per Equation (2) [64],

(2) $$Wo=\frac{\Delta \rho gV_{d}}{\pi \gamma D_{N}},$$

wherein, *Wo* is greater than 0.25. Herein, Wo captures the ratio of the gravity to the surface tension force, Δρ is the density difference between droplet and ambient fluid (i.e., air), $V_{d}$ is the maximum droplet volume attainable on the needle in the absence of any solute, γ is the effective surface tension, and $D_{N}$ is the needle diameter. A plot of the working range for the Worthington numbers used in this study (0.3–0.7) is presented in the Supporting Information, Figure S2. Further reduction in the droplet volume beyond a certain compression limit led to the collapse of the particle monolayer.

### 2.6. Estimation of the Interparticle Interaction Energy

In order to obtain an understanding of the link between the attributes of the particles used in this study and the nature of particle network morphology formed at the air–water interface, different inter-particle interactions were estimated as follows.

The screened Coulomb potential ($U_{Coulombic}$) between portion of the interfacially trapped particles suspended in the aqueous solution is calculated using Equation (3) [65,66],

(3) $$U_{Coulombic}=\frac{A}{r}e^{-\kappa r},$$

where r is the center-to-center interparticle distance. The pre-factor A is defined as per Equation (4) [66],

(4) $$A=\frac{Q_{eff}^{2}}{4\pi \varepsilon \varepsilon_{0}},$$

where $\varepsilon_{0}\;$is the permittivity of vacuum, ε is the permittivity of water, and $Q_{eff\;}$is related to the particle charge density, as per Equation (5) [66],

(5) $$\sigma =\frac{Q_{eff}}{\pi R^{2}{sin}^{2}(\theta )}$$

here, R is the particle radius and θ is the particle contact angle at the interface.

The interparticle dipolar repulsive interaction through the water phase, which originates from the asymmetric distribution of the ionic cloud formed around the portion of the particles submerged in water, was determined using Equation (6) [67].

(6) $$U_{dipolar}=\frac{q}{2\pi \varepsilon_{0}\varepsilon }\frac{1}{r}\left(\frac{\varepsilon_{1}}{\varepsilon }\frac{1}{{\left(\kappa r\right)}^{2}}\right),$$

where $\varepsilon_{1}\;$is the permittivity of air, q is the total charge present on the portion of the particle in contact with the water phase and is related to the particle charge density σ as $q=\sigma A_{w}$, where $A_{w}$ is the surface area of the particle exposed to the water phase.

The van der Waals interaction between two interfacially bound particles is determined as per Equation (7) [68],

(7) $$U_{vdW}=\frac{-A_{eff}R}{12D}$$

where D is the surface-to-surface interparticle distance and$\;\;A_{eff}$ is the effective Hamaker constant. For particles partially immersed at a fluid interface, the value of the Hamaker constant can be estimated as per Equation (8) [69],

(8) $$A_{eff}=A_{pp}+f^{2}\left(3-2f\right)\left(A_{pwp}-A_{pp}\right),$$

where $A_{pp\;}$is the Hamaker constant for particles in vacuo, $A_{pwp}\;$is the Hamaker constant for particles interacting through bulk water phase, and f is the fractional immersion of particles at the interface.

The heterogeneities present on the surface of the particle cause undulation of the three-phase contact line, which results in the elevation or depression of the fluid interface, leading to capillary interactions. The resulting capillary interactions can be calculated using Equation (9) [70] as follows,

(9) $$U_{cap}=-12\pi \gamma_{0}H^{2}cos\;[2\left(\varphi_{A}+\varphi_{B}\right)]\frac{r_{c}^{4}}{r^{4}}$$

where$\;\gamma_{0}\;$is the air–water surface tension, H is the amplitude of the contact line undulation, $\varphi_{A}\;$and $\varphi_{B}$ represent the orientation of particles defined with respect to the line joining the particles, and $r_{c}$ is the radius of the three-phase contact line. Details on the parameters used in the calculation of each interparticle interaction are provided in the Supporting Information.

### 2.7. Statistical Analysis

In order to assess the impact of particle size on the measured interfacial properties, statistical analysis was performed on the collected data. Based on a set of 3 trials, mean values and standard deviations were computed for different parameters. Contact angle of the droplet on different surfaces was reported after averaging both the right and left droplet angles in each trial. From the Langmuir trough experiments, an average inflection point area was obtained, which was used to calculate the static compressional modulus. The % of entrapped particles at the interface was determined from the surface pressure–area isotherms and inflection point data (see Supporting Information). The interfacial rheology measurements provided values for elastic and loss moduli, which were used subsequently to determine values for yield stress and critical strain. From the zeta potential measurements, values for zeta potential, mobility, and conductivity were obtained. These were then utilized to calculate the mean values of Debye length, charge density, and number of charges on particle surface. The standard deviation of these derived parameters was determined using the error propagation method. To determine the average particle radius from SEM measurements, 100 particles were analyzed in the images captured for each particle size.

## 3. Results and Discussion

### 3.1. Particle Wettability and Surface Charge

**Figure 1:**
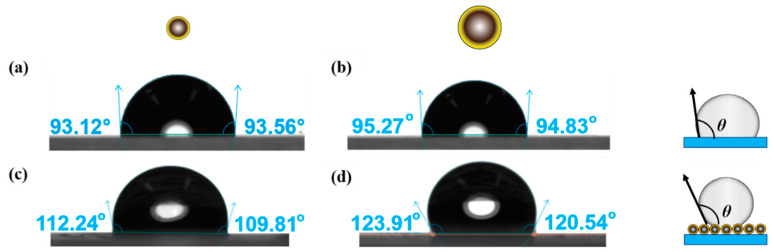
Contact angle determination for DCDMS-modified particles of two different sizes, 250 nm and 1000 nm. Images (**a**,**b**) represent a water droplet on silanized glass slides placed in the same reaction medium while modifying the 250 nm and 1000 nm particles, respectively. Images (**c**,**d**) illustrate a water droplet on a monolayer of hydrophobically modified 250 nm and 1000 nm particles coated on a glass substrate, respectively.

**Figure 2:**
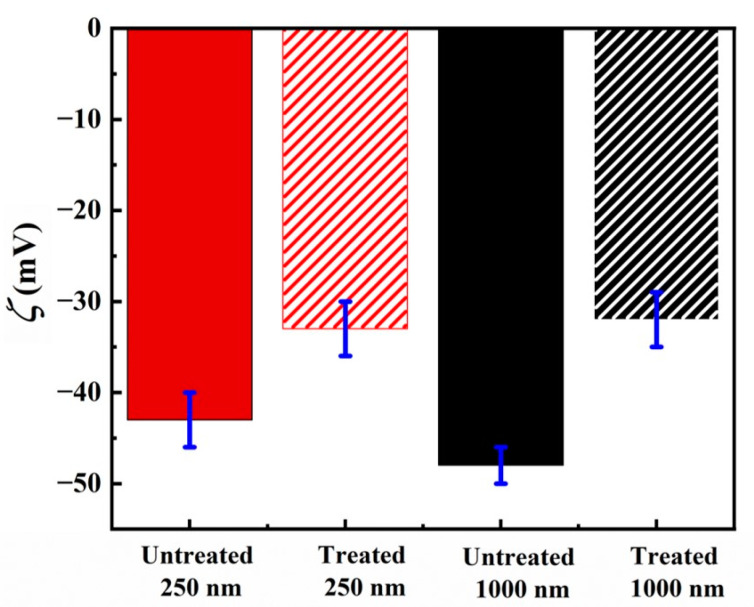
Zeta potential (ζ) measurements of silica particles of different size. Data belonging to 250 nm and 1000 nm particles are shown in black and red, respectively. Data for untreated particles are shown as solid bars and results obtained for the treated particles are displayed in the hashed bars.

The results of the contact angle (θ) measurements are provided in Figure 1 for water droplets deposited on the silanized glass slide (Figure 1a,b) and on a particle-coated glass substrate (Figure 1c,d). The contact angle values are similar for silanized glass substrates in both cases, θ = 93° ± 2° and 95° ± 2°, resulting from modification of the slides in the same media used for the treatment of 250 nm and 1000 nm particles, respectively. This result was expected considering that the same amount of silane was employed during the surface modification of the two particle sizes. Measuring the apparent contact angle of the water droplet on particle-coated substrates yields larger values compared to those obtained on glass substrates because of the roughness embedded onto the substrate in the presence of particles, which is known to amplify the wettability of the surface [71,72]. Given that the height and center-to-center distance of surface features have been established to affect the apparent contact angle on textured surfaces [72,73], and considering the resemblance between the two particle surfaces after silane treatment (refer to analysis in Figure 2), the variations observed in the measured apparent contact angle for the two particle-coated substrates can be attributed to the level of roughness present on each surface. This roughness may differ based on factors, such as the particle size, interparticle distance within the network, and the particle packing density on the surface.

Zeta potential measurements performed on both the untreated and treated samples for particles of different sizes, suspended in water, are provided in Figure 2. For the untreated particles, the zeta potential is measured to be −43 ± 3 mV and −48 ± 2 mV for 250 nm and 1000 nm particles, respectively. Once the particles are hydrophobically modified, the charged silanol groups present on the particle surface are replaced by the silane molecules, the grafting density of which also plays a role towards the stability of particles in the solution [74]. Upon silane modification, the value of zeta potential is reduced to −33 ± 3 mV for treated 250 nm particles and −32 ± 3 mV for 1000 nm particles. Based on the measured values of zeta potential, before and after silane treatment, the particle surface charge density can be calculated for both particle sizes. The differences in the surface charge density before and after the silane treatment can then be used to estimate the number of silanol groups removed from the particle surface, and replaced by the silane molecules, as a result of the surface modification. Details of the calculations are provided in the Supporting Information (Table S1), and the results of this analysis are provided in Table 1. As can be seen, the calculated value for the number of charges replaced on the particle surface is similar for both particle sizes (i.e., 0.012 µC/cm^2^ for 250 nm particles and 0.010 µC/cm^2^ for 1000 nm particles). Thus, from the results of the surface characterizations carried out on both particles, it could be inferred that their silane grafting density is comparable.

### 3.2. Langmuir Trough Studies

**Figure 3:**
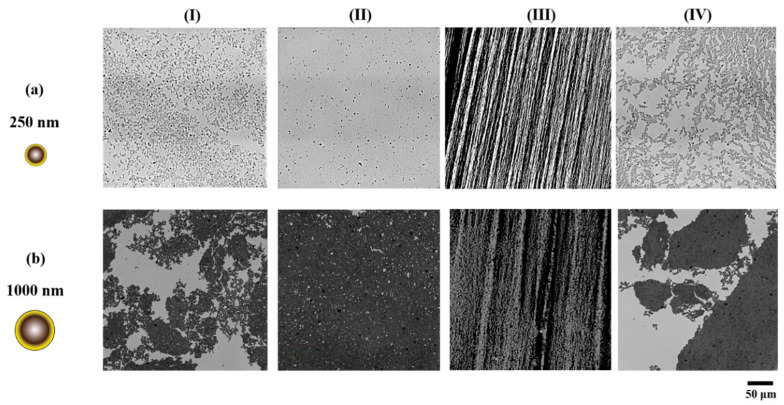
Optical microscopy images for particle networks formed by (**a**) 250 nm and (**b**) 1000 nm hydrophobically modified silica particles during a compression–expansion cycle. Panels (**I**) to (**IV**) display images captured at various stages of the compression–expansion cycle as follows: (**I**) open-barrier state upon particle deposition, (**II**) maximum packing in 2D, (**III**) collapse via buckling, and (**IV**) fully expanded state. Scale bar is 50 µm.

**Figure 4:**
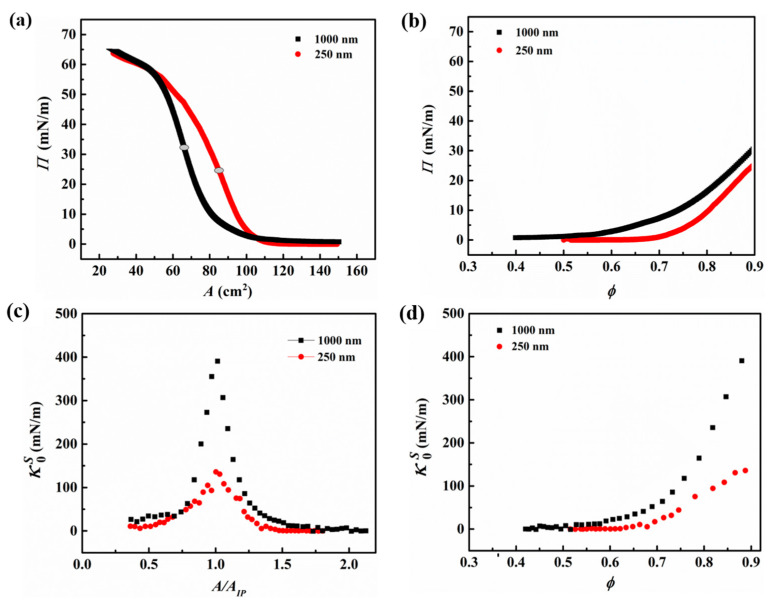
Data on Langmuir trough experiments carried out on 250 nm (red circles) and 1000 nm (black squares) particles. (**a**) Surface pressure as a function of trough area (A), where the highlighted points indicate the inflection point ($A_{IP}$) of the isotherms, (**b**) surface pressure as a function of particle surface coverage (ϕ), (**c**) static compressional modulus of the interfacial network as a function of trough area normalized by the area at the inflection point of the isotherm, and (**d**) static compressional modulus of the network as a function of surface coverage of two particle sizes.

The microstructure of interfacial monolayers formed by the hydrophobically modified silica particles and the changes associated with the corresponding surface pressures during the applied compressions are analyzed using a Langmuir trough. The rearrangements occurring within the network during the compression and expansion of the monolayer, captured via an optical microscope, are presented in Figure 3, and the accompanying surface pressure isotherms are provided in Figure 4a. Upon the deposition of particles (i.e., open-barrier state), the particle arrangement on the interface is different for the two sizes; while the 250 nm particles create an open network composed of individual particles and small clusters, the 1000 nm particles form large interfacial aggregates (see Figure 3, panel I). As can be seen in Figure 4a, the initial surface pressure upon the particle deposition remains below 1 mN/m in both cases. Upon the compression of the monolayer and densification of the particles at the interface, the surface pressure increases until the monolayer has reached a maximum packing in 2-dimensions, as captured in micrographs of Figure 3, panel II. The inflection point of the surface pressure isotherm is shown to correspond to the maximum packing of the interfacial layer [42,75]. As marked in Figure 4a, the inflection point of the 250 nm particle networks occurred at 25 ± 2 mN/m, while for 1000 nm, it corresponded to a surface pressure of 32 ± 1 mN/m, averaged over three trials. Table S2, further highlights the high degree of particle entrapment at the interface, for both particle sizes, as determined from the inflection point of the isotherms. Compression of the interfacial network beyond this state leads to stress relaxation in the form of buckling of the particle layer, as captured in the micrographs of Figure 3, panel III, in agreement with the previously reported collapse mode for hydrophobic particles [42,58,76]. Upon reaching the closed-barrier state (i.e., 25 cm^2^), both particle networks attained similar surface pressure values. The barriers are then subsequently opened, and the network is allowed to relax. Figure 3, panel IV, depicts the final microstructure of the particle networks resulting from a full compression–expansion cycle. While the interfacial network of the 250 nm particles disintegrated into smaller clusters upon expansion, the 1000 nm particles remained within larger aggregates.

To compare the impact of particle size and interparticle interactions on the resulting surface pressures, at similar values of particle surface coverage (ϕ) for the two sizes, the inflection point of the isotherm marked in Figure 4a is used to estimate the surface coverage along the isotherm, the results of which are plotted in Figure 4b. The rise in the surface pressure is observed to occur at a lower value of surface coverage for the 1000 nm particles (ϕ~0.55) in comparison to the case of 250 nm particles (ϕ~0.7), which could be attributed to stronger interparticle interactions in the former case, as corroborated by the microscopy images of the network (panel I in Figure 3). Moreover, it can be inferred that the network formed by the larger particles exhibits a higher surface pressure when compared to the case of smaller particles at the same surface coverage, which could arise from their stronger interparticle interactions.

The strength of the interfacial networks formed by the two particle sizes can be captured in their static compressional modulus ($\kappa_{0}^{s}$), presented in Figure 4c as a function of the normalized trough area $A/A_{IP}$, where A is the trough area and $A_{IP}$ is the trough area at the inflection point marked in Figure 4a. The network of 1000 nm particles depicted a higher modulus value at each point during the compression, and it is more prominent at the point of maximum 2D packing with a more robust network, exhibiting a static modulus of 391 ± 34 mN/m compared to 140 ± 22 mN/m for 250 nm particles. The modulus of the network is also compared as a function of the surface coverage for the two particle sizes, as shown in Figure 4d. The data indicate that the enhancement in the modulus of the network that results from particle densification occurs at lower surface coverages for the larger particle network and rises more sharply as the surface coverage increases.

In order to investigate the response and stability of the particle network subjected to cyclic compressions and expansions, the hysteresis of surface pressure isotherms is studied by performing three compression–expansion cycles on the particle-laden interfaces, the results of which are provided in Figure S3. These measurements further illustrate that particle densification takes place during the first compression, for both sizes, which moves the pressure lift-off to a smaller trough area on the second compression, whereas no further changes in pressure response of the monolayers are captured between cycles two and three. Detailed analysis of the results obtained from the cyclic measurements can be found in the Supporting Information.

### 3.3. Shear Rheology of the Interfacial Particle Networks

**Figure 5:**
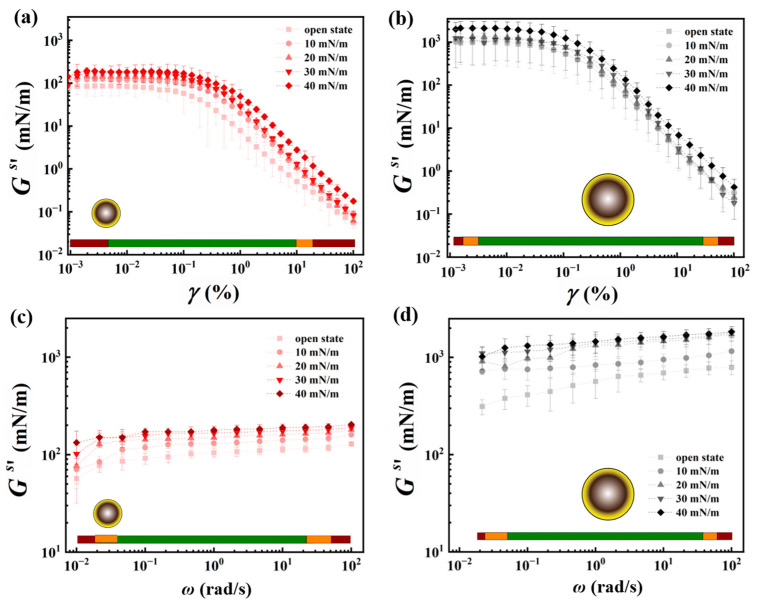
Rheological characterization of the 250 nm and 1000 nm particle networks at the air–water interface under oscillatory shear. (**a**,**b**) depict the amplitude sweep measurements; (**c**,**d**) depict the frequency sweep. The color coding in the bar placed at the bottom of the plots represents the reliability in operating windows while interpreting the data.

Data on the strain amplitude sweep of the interfacial networks, at various surface pressures, formed by 250 nm and 1000 nm particles, are displayed in Figure 5a,b**,** respectively. The operating window labelled in green corresponds to the measurement parameters that yield reproducible data and is indicative of the network characteristics. At the extreme ends of the strain values probed, the measured data are labeled as yellow and red, indicating that caution needs to be exercised while interpreting the data. Within the green range of applied strains, the characteristic linear viscoelastic region (LVR) can be identified from the response of the monolayer in the form of the plateau in the monolayer’s elastic surface modulus ($G^{s\prime }$). Upon increasing the strain, a reduction in the elastic modulus occurs, indicating the disruption of the microstructure at these higher strain values. The magnitude of the elastic modulus increases gradually with increasing the surface pressure, for both particle sizes, which can be attributed to the formation of an interconnected compact network at higher surface coverages. $G^{s\prime }$ reaches its maximum value as the particle network approaches its maximum packing fraction (i.e., surface pressures in proximity to the inflection point of the isotherm determined from Figure 4a). The elastic modulus exhibited by the densest interfacial network of 250 nm particles is 170 ± 40 mN/m, compared to 1100 ± 30 mN/m for the particle network formed by the 1000 nm particles. Furthermore, for all particle networks, the measured values of storage moduli ($G^{s\prime })\;$are higher than the loss moduli ($G^{s\prime \prime }$), representing the elastic-like nature of the interfacial monolayers formed by the hydrophobically modified silica particles. Data on viscous moduli of the network at various surface pressures are provided in Figure S4.

Data obtained from the amplitude sweep measurements provided information on the LVR of the interfacial microstructures. Based on this information, a strain amplitude within the LVR is chosen at which the frequency sweep measurements are carried out. Results of the small-angle oscillatory shear measurements performed with 0.01% strain and in a frequency range of 0.01–100 rad/s are provided in Figure 5c,d for the interfacial networks formed by 250 nm and 1000 nm particles, respectively. The corresponding loss modulus data and the loss tangent data are provided in the Supporting Information, Figure S5. The results obtained in a frequency window of 0.1–10 rad/s are in the green range and indicative of the network characteristics. In agreement with the amplitude sweep results, it is observed that the $G^{s\prime }$ values are almost an order of magnitude higher for the 1000 nm particles compared to those obtained for the 250 nm sample at equivalent surface pressures. At low surface pressures of 10 mN/m and below, the elastic modulus is observed to increase with frequency, which could be attributed to a network formed at moderate surface coverage of particles (see Figure 4b) that allows for rearrangement and reorganization of particle clusters present at the interface. This frequency dependency is observed to decrease with increasing the surface pressure and approaching maximum close packing at the interface, as the formation of large aggregates may prevent particle rearrangement within the time span corresponding to the applied frequencies at such high surface coverages. As the inflection point of the particle network is estimated to be 25 ± 2 mN/m for the 250 nm particles and 32 ± 1 mN/m for the 1000 nm particles, it could be inferred that the jammed structure obtained at high surface coverage of particles caused the formation of a rigid-like network. It may also be noted that the variation in the surface shear elastic modulus across the different surface pressures is larger for the 1000 nm particles in comparison to that of the 250 nm particle network. This may have resulted from the lower surface coverages needed for the larger particles to attain the same surface pressures (see Figure 4b), allowing for more particle rearrangements. In the low-frequency range, between 0.01 rad/s and 0.1 rad/s, the obtained data fell in the “red” and “yellow” ranges due to the instrument limitations and, hence, are to be used with caution. Similarly, the data obtained in the frequency range of 10–100 rad/s fell mostly in the “yellow” and “red” ranges, as the data could have been affected by the instrument inertia.

**Figure 6:**
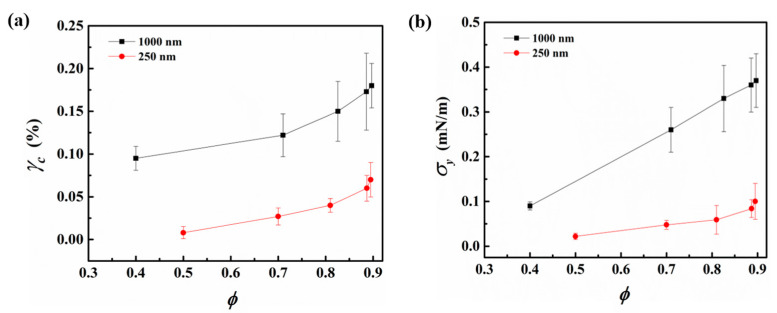
(**a**) Critical strain ($\gamma_{c}$) and (**b**) yield stress ($\sigma_{y}$) of interfacial networks formed by 250 nm and 1000 nm particle at the air–water interface, respectively, as a function of particle surface coverage.

The amplitude sweep data shown in Figure 5a,b can be further analyzed to obtain the critical strain ($\gamma_{c}$) of the interfacial networks, which reflects the amount of shear strain that can be sustained by the interfacial microstructure formed by the particles above which the material flows. The yield stress ($\sigma_{y}$) of the network can then be calculated as per Equation (10) [35],

(10) $$\sigma_{y}=G_{c}^{s\prime }\gamma_{c},$$

where $G_{c}^{s\prime }$ is the plateau value of the surface elastic modulus at shear strains below $\gamma_{c}$. The determination of the critical shear strain and the calculation of yield stress were carried out following the work of Beltramo and coworkers [35]. From Figure 6a, it can be seen that the value of critical strain that can be sustained by the interfacial networks increases with the surface coverage for both particle networks; however, the magnitude of critical strain is higher in the case of 1000 nm particles. The initial gradual increase in the critical strain values, at moderate surface coverage of particles, can be attributed to the densification of the particle network. With increasing the surface pressure, corresponding to an increase in surface coverage, the rate of increase in critical strain reduces as the jamming of the particle network is approached.

Figure 6b presents the yield stress values for networks formed by the 250 nm and 1000 nm particles at various surface coverages. It can be noted that the magnitude of $\sigma_{y}\;$for the 1000 nm particle network is larger than 250 nm and that the enhancement in the yield stress with an increase in the particle surface coverage is larger for the case of 1000 nm particles, as compared to the 250 nm particles.

### 3.4. Dilational Rheology of Interfacial Particle Networks

**Figure 7:**
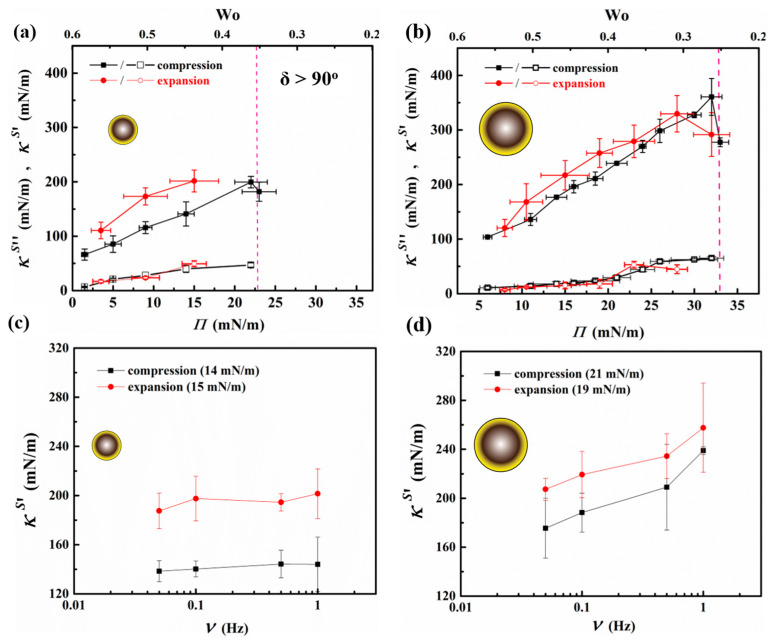
Results of dilational rheology measurements carried out on particle-laden pendant droplets comprising 250 nm and 1000 nm particle networks. (**a**,**b**) Elastic ($\kappa^{s\prime }$, filled symbol) and loss ($\kappa^{s\prime \prime }$, open symbol) surface moduli values of the networks, measured using oscillating frequency of 1 Hz, at different surface pressures reached during the compression and expansion segments of a cycle. The working Worthington number over which the data are obtained is provided on the plots. The vertical dashed lines on the plots indicate the surface pressure beyond which the phase shift is δ > 90°. (**c**,**d**) Variation in the elastic surface modulus ($\kappa^{s\prime }$) with frequency of oscillations measured at two similar surface coverages for 250 nm and 1000 nm particle networks.

Figure 7a,b present the dilatational surface elastic ($\kappa^{s\prime }$) and viscous ($\kappa^{s\prime \prime }$) moduli of interfacial networks formed by the 250 nm and 1000 nm particles measured at different surface pressures during a compression and expansion cycle. The Wo numbers corresponding to these measurements are also provided along with the data.

It is observed from the plot that a rise in the magnitude of the surface elastic modulus is directly proportional to the surface pressure generated in the presence of particles on the droplet surface, in agreement with work by Johnston et al., where a similar rise in surface elastic dilational modulus was reported via the addition of sulfonated particles to the interface [77]. Upon reducing the droplet volume and compressing its surface, the coverage of particles on the droplet surface increases, particle clusters begin to form, and their interaction results in an increased surface pressure and the creation of a particle network with a higher surface elastic modulus. While oscillating at 1 Hz frequency, the particle-laden droplet exhibits a viscoelastic response with the surface elastic modulus dominating the behavior, where $\kappa^{s\prime }\;$value increases gradually with surface pressure until it reaches a critical value. At this junction, the dense particulate network generates an elastic response with the highest value of $\kappa^{s\prime }$, which could be attributed to strong interparticle interactions within the monolayer. The observations are similar to results obtained from experiments performed on the Langmuir trough, where, with rising surface coverage, the static compressional modulus increased and reached its highest value at the maximum close packing of particles in 2D (see Figure 4c). The inflection point of the isotherm, obtained from the Langmuir trough experiments, is comparable to the surface pressure value achieved in drop tensiometry corresponding to the maximum $\kappa^{s\prime }$ measured (199 ± 11 mN/m for 250 nm particles and 359 ± 34 mN/m for 1000 nm particles, respectively). Compressing the droplet beyond this surface pressure results in the collapse of the monolayer via buckling, where the oscillations result in phase shift δ > 90° and correspond to Wo values close to the lower-bound limit.

Expanding the pendant drop by injecting water back into the compressed droplet results in the recovery of the network and its surface elastic modulus. It is observed that at equivalent surface pressures, the $\kappa^{s\prime }$ values obtained during the expansion leg of the cycle are larger than those measured during the compression segment of the cycle for interfacial networks formed by both particle sizes, as depicted in Figure 7a,b. This behavior could be attributed to particle densification during the compression that results in an already existing interfacial network at the beginning of oscillatory experiments on the expansion leg of the cycle. It may be noted that, for the case of a network formed by the larger particles, the $\kappa^{s\prime }$ values obtained are higher in magnitude than the smaller-particle network exhibiting the same surface pressure. Moreover, the larger particle network can sustain a higher surface pressure, exhibiting a larger elastic modulus prior to buckling.

Figure 7c,d show the influence of oscillation frequency on the response of the interfacial networks formed by the 250 nm and 1000 nm particles, respectively. The value of surface pressures chosen to demonstrate the effect of applied frequency on the measured $\kappa^{s\prime }$ values is based on comparable surface packing between the two particle sizes; a surface coverage of ϕ~0.82 corresponds to surface pressures of Π~15 mN/m and Π~20 mN/m for 250 nm and 1000 nm particle networks, respectively, according to the Langmuir trough measurements depicted in Figure 4b. In agreement with the results obtained at a frequency of 1 Hz, shown in Figure 7a,b, the surface elastic modulus measured during the expansion leg of the cycle is higher than that acquired on the compression, for both particle types and across the frequency ranges studied. In addition, the network formed by the 1000 nm particles exhibits a larger frequency-dependent response compared to that of the 250 nm particle network, where increasing the frequency of oscillations leads to an enhancement in the modulus of the network.

### 3.5. Analysis of Interparticle Interactions

**Figure 8:**
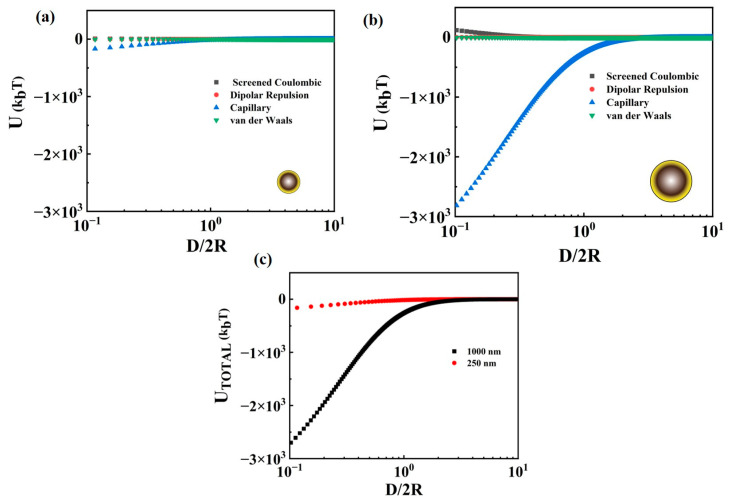
Change in the magnitude of various interparticle interactions at play with the normalized distance (D/2R) for (**a**) 250 nm and (**b**) 1000 nm particles at the air–water interface; D is particle surface-to-surface distance and R is the particle radius. (**c**) The sum of all interactions ($U_{TOTAL}$) is depicted in (**a**,**b**) for the two different size particles.

To better understand the impact of particle attributes on the observed microstructures and the measured rheological responses detailed above, we analyzed the interparticle interactions resulting from a pair of particles interfacially bound to the air–water interface. To provide an insight towards the characteristics of the network formed by the respective particles, we determined the total interparticle interaction potential ($U_{TOTAL}$) by examining the screened coulombic interactions ($U_{Coulombic}$) and the dipolar repulsion ($U_{dipolar}$) through the water phase, the van der Waals interaction through both phases ($U_{vdW}$), and the capillary interactions between the interfacially trapped particles ($U_{cap}$); the results are shown in Figure 8a,b for a pair of 250 nm and 1000 nm particles, respectively. Figure 8c presents the change in the total interparticle interaction as a function of the surface-to-surface separation distance between the particles (D) normalized by the average particle size (i.e., 2R), as measured using SEM. It can be observed that while the attractive interparticle interactions dominate over the repulsive interactions for both sizes, the magnitude of the total interaction is higher for the 1000 nm particles compared to the 250 nm particles. As the term D/2R approaches 1, i.e., the interparticle distance is of the order of the particle dimension, the magnitude of the total interaction potential increases; $U_{TOTAL}$ is ~311 $k_{B}T\;$for 1000 nm particles, whereas it is ~41 $k_{B}T\;$for 250 nm particles, with $k_{B}T\;$as the thermal energy scale. The total interaction energy is overall attractive in both cases at D/2R~1. The larger value of $U_{TOTAL}$ for 1000 nm particles reflects the stronger interaction between these particles that engendered a robust network and complements the results obtained on the microstructure of the resulting networks, which showed a network with bigger aggregate size for 1000 nm particles. The stronger interparticle interaction for the 1000 nm particles is also in line with the obtained rheological data for the network formed by these particles that exhibited a higher yield strain and larger surface elastic modulus when compared to the 250 nm particles at a similar surface coverage.

During the compression studies performed on the Langmuir trough, it was observed that the bigger particles formed networks comprising large aggregates, whereas the smaller particles yielded smaller mostly disconnected clusters. Moreover, the higher value of surface pressure at the same ϕ (Figure 4b) and higher $\kappa_{0}^{s}\;$reached at the inflection point of the isotherm (Figure 4c) are observed for the larger particle network, which could be attributed to the presence of dominant attractive capillary interactions. The difference in interparticle interactions was also evident when the monolayer underwent expansion; while disconnected islands resulted from the expansion of the 250 nm particle network, relatively larger-sized clusters were obtained by expanding the network obtained with the 1000 nm particles, as shown in Figure 3. The particle networks also displayed similar characteristics when subjected to oscillatory shear measurements, where larger particle networks demonstrated higher values of $\gamma_{c}$ and $\sigma_{y}\;$when compared to those obtained for networks of smaller particles at similar surface coverages. The attractive interactions may have allowed the larger particle network to resist deformation more strongly compared to the smaller particle network. Similar observations were also made during the dilatational analysis, wherein the results obtained complemented the findings obtained via Langmuir trough studies and shear measurements.

While this research study focused on two specific particle sizes (250 nm and 1000 nm), the findings obtained can be applied to examine the impact of size in networks formed by particles exhibiting similar surface attributes (e.g., charge density, surface chemistry, contact angle at the interface). It is important to note that for significantly smaller particles (a few nanometers in size), the binding energy becomes comparable to the thermal energy scale, necessitating consideration of reversible adsorption and desorption of particles to the fluid interface. Conversely, for significantly larger particles (ten microns in size), the weight-induced deformation of the fluid interface gives rise to monopolar capillary interactions, which should also be taken into consideration.

## 4. Conclusions

The effects of particle size on the microstructure of the network formed at the air–water interface and its rheological properties were investigated using hydrophobically modified silica particles. Analysis of pair interactions at the interface indicated that the larger particles possessed stronger interparticle interactions compared to the smaller particles. The difference in the interparticle interactions across the two particle sizes manifested itself in the microstructure of the resulting interfacial networks and the mechanical properties measured via various interfacial rheological tools. Upon subjecting the particle networks formed at the air–water interface to compressional stress on a Langmuir trough, the larger particle network exhibited a higher static compressional modulus and a higher surface pressure when compared to the smaller particles at the same surface coverage. A similar trend was observed in the oscillatory rheological measurements. Strain amplitude sweep measurements in shear mode indicated a higher critical yield strain and yield stress for the larger particle network, while during the frequency sweep analysis, the larger particle networks demonstrated a higher value of shear modulus. Pendant tensiometry results showed that the larger particle networks yielded a higher elastic modulus compared to smaller particles at the same surface coverage. Although for the smaller particle network, the elastic surface modulus did not vary much with the frequency of applied oscillations, the larger particle network displayed a frequency dependency, where the surface modulus enhanced with the droplet actuation frequency. The results obtained in this work highlight the impact particle attributes including size could have on the interparticle interactions that subsequently determine the interfacial microstructure and mechanical robustness of particle-laden interfaces when subjected to shear and dilatational stress. The findings of the conducted study could be beneficial in the design of complex interfacial systems, especially in applications involving flows, such as froth flotation processes, recovery of rare-earth elements via surfactant/nanoparticle complexes, foams for subsurface energy recovery, and emulsions for the food industry.

## Figures and Tables

**Table 1:** Analysis of the treated and untreated particles based on the data obtained from the zeta potential measurements.

Particle Attribute ⇒	ζ (mV)	σ (μC/cm^2^)
Particle Type ⇓		
Untreated 250 nm	−43 ± 3	0.074 ± 0.010
Treated 250 nm	−33 ± 3	0.062 ± 0.007
Untreated 1000 nm	−48 ± 2	0.051 ± 0.003
Treated 1000 nm	−32 ± 3	0.041 ± 0.003

## Data Availability

All data that support the findings of this study are included within the article (and any Supplementary Files).

## Supplementary Materials

The following supporting information can be downloaded at: https://www.mdpi.com/article/10.3390/nano13142114/s1, Figure S1: Zeta potential measurements for untreated and silane-modified 250 nm and 1000 nm particles in ethanol; Table S1: Characteristics of the untreated and treated particles obtained from zeta potential analysis. Parameters shown are as follows: mobility (μ), zeta potential (ζ), solution conductance (χ), Debye length ($\kappa^{-1}$), Henry’s function f(κR), surface charge density σ, and number of charges present on the particles surface; Figure S2: Working limits for the tensiometry experiments presented as a function of droplet area and Wo number as a function of droplet volume. The cyan region corresponds to the working range wherein the data obtained was utilized for analysis presented in the main text. The magenta region corresponds to the operating window falling in the low Wo region, where the pendant drop shape can no longer be defined using Young-Laplace equation and the results obtained have a large error associated with them; Table S2: Determination of trapping efficacy for both particle sizes; Figure S3: Plot depicting Hysteresis of interfacial particle networks examined via three successive compression–expansion cycles carried out on samples of (a) 250 nm and (b) 1000 nm particles at the air–water interface; Figure S4: Complex modulus values for the different treated silica nanoparticle networks obtained during the amplitude sweep measurements. Plots (a) and (b) present the elastic modulus data and plots (c) and (d) represent the viscous modulus data for 250 nm and 1000 nm particle types, respectively; Figure S5: Rheological properties of the treated silica nanoparticle networks obtained via frequency sweep measurements. (a) and (b) display the elastic modulus of the networks present at different surface pressures, for 250 nm and 1000 nm particles, respectively; (c) and (d) illustrate the viscous modulus of the networks present at different surface pressures for 250 nm and 1000 nm particles, respectively; (e) and (f) present the elastic modulus, viscous modulus, and the loss tangent data for networks at surface pressure of 30 mN/m, and for 250 nm and 1000 nm particles, respectively. (See Refs. [51,62,78,79,80,81,82]).
