# Supplementary material for: Particle Size and Rheology of Silica Particle Networks at the Air–Water Interface
Source: Nanomaterials (Basel). 2023 Jul 20;13(14):2114. doi: 10.3390/nano13142114 (PMC10386461; doi:10.3390/nano13142114)
Supplement: Supplementary file 1 [file nanomaterials-13-02114-s001.zip › nanomaterials-2501263-supplementary.pdf]

nanomaterials

Supplementary Information

Particle Size and Rheology of Silica Particle Networks at the Air-Water Interface

Siddharth Thakur and Sepideh Razavi

School of Sustainable Chemical, Biological and Materials Engineering, University of Oklahoma, Norman, OK, USA

Parameters used in the calculation of zeta potential

The conductance values obtained from the mobility measurements are employed to calculate the effective ionic concentration, as per $\chi = C_i \Lambda^0 \times 10^9$, where C_i is ionic concentration, Λ^0 is molar ionic conductivity, and χ is conductance. It may be noted that the values for molar ionic conductivity (S.cm^2.mol^{-1}) taken for different ions were as follows: H^+ (349), OH^- (198).[1] These parameters are then taken into consideration for determination of Debye length (κ^{-1}) as per $\kappa^2 = \frac{2C_i(ze)^2}{\varepsilon_0 \varepsilon k_B T}$, where z is the ion valency, e is the electron charge, ε_0 is the permittivity of vacuum, ε is the permittivity of water, k_B is Boltzmann constant, and T is temperature.[2] Once the relative thickness of Debye length with regards to particle radius is known, a Henry's correction factor, $f(\kappa R)$,[3] is determined and utilized to calculate the particles zeta potential according to $\mu = \frac{2\varepsilon_0 \varepsilon \zeta}{3\eta} f(\kappa R)$, where η is the viscosity of water (0.001), μ is the measured mobility, and ζ is the calculated zeta potential. Next, surface charge density on the particle can be determined as per $\sigma = \varepsilon_0 \varepsilon \kappa \Psi_0 [\frac{1+\kappa R}{\kappa R}]$, where Ψ_0, is the electrostatic surface potential, approximated as the zeta potential, and σ is the particles surface charge density.[4]

Zeta potential measurements of the untreated and treated silica nanoparticles of both particle sizes are also carried out in ethanol as depicted in **Figure S1**. For the untreated particles, the measured zeta potential is –46 ± 4 mV for 250 nm compared to –49 ± 3 mV for 1000 nm. After treatment, the values of the zeta potential measurements are reduced to –38 ± 3 mV and –37 ± 3 mV for 250 nm and 1000 nm particles, respectively.

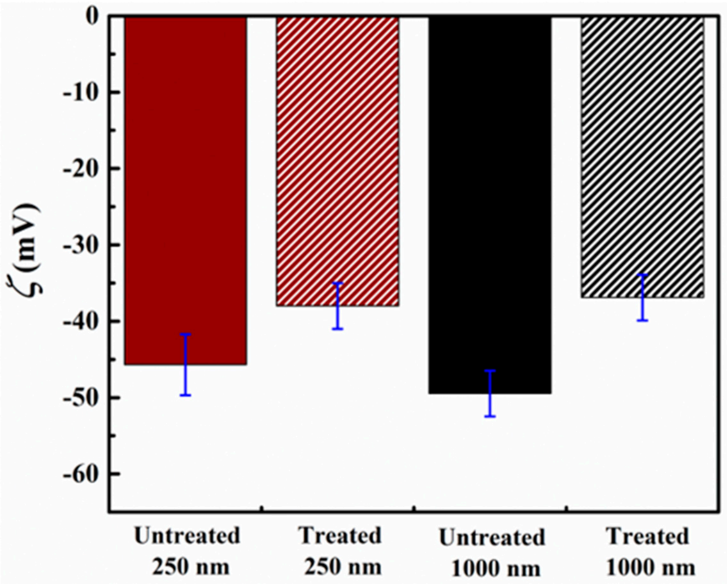

Figure S1. Zeta potential measurements for untreated and silane-modified 250 nm and 1000 nm particles in ethanol.

Particle surface charge density and surface modification

Table S1 provides the data reflecting the changes in the particle surface attributes that resulted from the silane treatment and are obtained from mobility measurements. It also provides other parameters such as conductance, Debye length, and Henry's function that were utilized to calculate the surface charge density values. Total charge on the particle surface was obtained from the product of surface charge density and total surface area of each nanoparticle ($4\pi R^2$), where R is the average particle diameter determined from the SEM measurements (values of 133.5 ± 8.5 nm and 493.5 ± 10.7 nm, were used for the 250 nm and 1000 nm particles, respectively). Number of surface charges replaced upon silane

modification was obtained from the difference between the surface charge densities of the treated and untreated particles calculated based on the determined zeta potential values.

Table S1. Characteristics of the untreated and treated particles obtained from zeta potential analysis. Parameters shown are as follows: mobility (μ), zeta potential (ζ), solution conductance (χ), Debye length (κ^{-1}), Henry's function $f(\kappa R)$, surface charge density (σ), and number of charges present on the particles surface.

**Particle & solution attributes** ⇒ **Particle Type** ⇓	ζ **Copyright:** © 2023 by the authors. Licensee MDPI, Basel, Switzerland. This article is an open access article distributed under the terms and conditions of the Creative Commons Attribution (CC BY) license (https://creativecommons.org/licenses/by/4.0/). **(mV)**	μ **(μm/s)/(V/cm)**	χ **(μS)**	κ^{-1} **(nm)**	$f(\kappa R)$	σ **(μC/cm²)**	**# of charges**
Untreated 250 nm	− 43 ± 3	-3.3 ± 0.4	3.4 ± 0.4	123.0 ± 15.1	1.03 ± 0.06	0.074 ± 0.010	1041.4 ± 158.2
Treated 250 nm	− 33 ± 3	-2.8 ± 0.4	3.5 ± 1.0	120.4 ± 34.4	1.03 ± 0.06	0.062 ± 0.007	865.7 ± 112.5
Untreated 1000 nm	− 48 ± 2	-3.7 ± 0.2	3.4 ± 0.4	121.7 ± 13.9	1.16 ± 0.07	0.051 ± 0.003	9788.6 ± 890.5
Treated 1000 nm	− 32 ± 3	-3.2 ± 0.1	3.4 ± 1.0	120.7 ± 35.0	1.13 ± 0.07	0.041 ± 0.003	7831.2 ± 712.9

Operating window for oscillatory shear measurements

In order to capture the physics pertaining to interfacial networks by extracting information from oscillatory shear measurements, a balance needs to be struck between the signal to noise ratio and the influence of geometry interia. To identify the data that fall in this region, a color-coded guide is presented based on the conditions determined from the work of Vermant and coworkers[5] as follows. The strain/frequency range is labelled as green if, (I) the measured oscillatory stress is at least 10 times larger than the oscillatory stress related to the geometry, (II) measured oscillatory torque is atleast 10 times larger than the minimum torque limit of the rheometer (0.1 μN.m), and (III) the raw phase angle is smaller than 90°. Similarily, the strain/frequency range is labelled as yellow if the conditions (I) and (II) have ratio ≥ 5, raw phase angle is smaller than 90°, while it is labelled as red for all other conditions. The values of parameters needed for determining the operating window in shear rheolgy experiments such as oscillatory stress related to geometry, minimum torque limit, and raw phase angle can be obtained from the instrument. For instance, for condition

(I), the oscillatory stress related to geometry is determined as per the formula: $|G_s^*| > \frac{C_M}{C_\theta} I\omega^2$, where G_s^* is the complex viscoelastic modulus (N/m), I is the sum of geometry and instrument inertia (2.3×10^{-5} N m.s^2), ω is the driving frequency (rad/s, e.g., the frequency range used in the sweep measurements is 10^{-2} to 10^2 rad/s), C_θ is the geometric factor that relates to the conversion of displacement (5.21), θ, to the interfacial strain, C_M is the factor that concerns the conversion of torque to interfacial stress (138.1 m^{-2}). The values for these parameters, that is I, C_θ, C_M are provided by the instrument (values presented are obtained from the rheometer used for this study).

Working range for tensiometry measurements

Figure S2 depicts the corresponding change in area and respective value of the *Wo* number for droplet volumes used in tensiometry experiments with 1000 nm particles. To obtain reliable readings, the tensiometry analysis is performed on droplet volume in the range of 44 ± 2 µL to 20 ± 2 µL (cyan color in **Figure S2**).

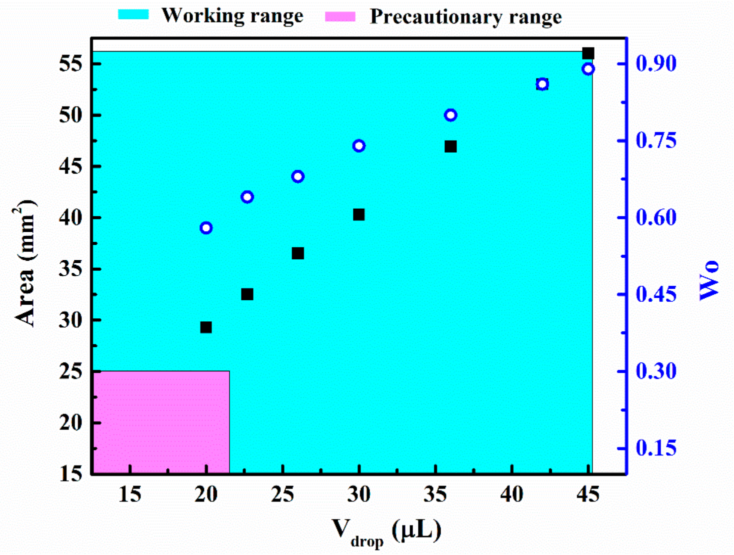

Figure S2. Working limits for the tensiometry experiments presented as a function of droplet area (solid symbol) and *Wo* number (open symbol) as a function of droplet volume. The cyan region corresponds to the working range wherein the data obtained was utilized for analysis presented in the main text. The magenta region corresponds to the operating window falling in the low *Wo* region, where the results obtained have a large error associated with them.

Hysteresis analysis on surface pressure isotherms

Table S2 represents the trapping efficiency of both particle sizes at the air-water interface. Using the same dispersion concentration of 37.5 mg/mL, and with deposition of 100 µL for 250 nm particles and 300 µL for the 1000 nm particles, the number of particles deposited at the interface can be calculated as shown in **Table S2**. It is understood that the number of particles deposited at the interface is higher for the smaller particle size sample. However, all the deposited particles will not necessarily get trapped at the interface and therefore there is a need to calculate an entrapment efficacy for each particle size. To do so, the surface pressure isotherms are used to locate the area corresponding to the inflection point (A_{IP}) at which point the maximum close packing is obtained at the interface. Since the surface coverage of particles are assumed to be $\phi = 0.89$ at the inflection point, using the A_{IP} and the cross-sectional area of particles, the total number of particles

trapped at the interface can be calculated. Both particles exhibit a high entrapment efficacy of value greater than 0.90.

Table S2. Determination of trapping efficacy for both particle sizes.

Size	# Particles Deposited	# Particles Trapped	Entrapment Efficacy
250 nm	$1.5\times10^{11} \pm 3.5\times10^{10}$	$1.4\times10^{11} \pm 1.9\times10^{10}$	0.93 ± 0.25
1000 nm	$8.4\times10^{9} \pm 4.6\times10^{8}$	$7.7\times10^{9} \pm 2.8\times10^{8}$	0.91 ± 0.06

Hysteresis of the particle networks at the air-water interface is investigated by performing cyclic compression-expansion experiments, results of which are provided in **Figure S3**. The first thing to be noted is that the final surface pressure attained for both sets of particles is similar across the three cycles. Hence, particle expulsion to the sub-phase during the cyclic compressions and expansions can be ruled out. As depicted in **Figure S3(a)**, for 250 nm particles, the pressure lifts off at a trough area of ~110 cm^2 during the first compression. After the monolayer is relaxed at the end of the 1st compression-expansion cycle, the particle network breaks up into small clusters and disconnected aggregates, as shown in **Figure 3(a)-IV**, which could be attributed to their relatively weak attractive interactions. As the 2nd cycle begins, the surface pressure lift-off is moved to a smaller trough area of ~50 cm^2 and a sharp rise in the surface pressure is recorded due to clustering that occurred during the 1st cycle. No further hysteresis is observed in the isotherms between the 2nd and 3rd cycles. Once the network has relaxed after the 1st cycle, the smaller aggregates from 250 nm particle network may allow for more rearrangement/reorganization and hence may lead to rise in surface pressure at smaller trough areas. In comparison, after the completion of the 1st cycle, the network formed by 1000 nm particles exhibits aggregates that are larger in size. Therefore, the monolayer exhibits a relatively more gradual rise in surface pressure, during the 2nd and 3rd compression, as seen in **Figure S3(b)**. For both particle sizes, during the subsequent compression cycles, the surface pressure lift-off takes place at higher trough areas owing to the monolayer densification during the first compression.

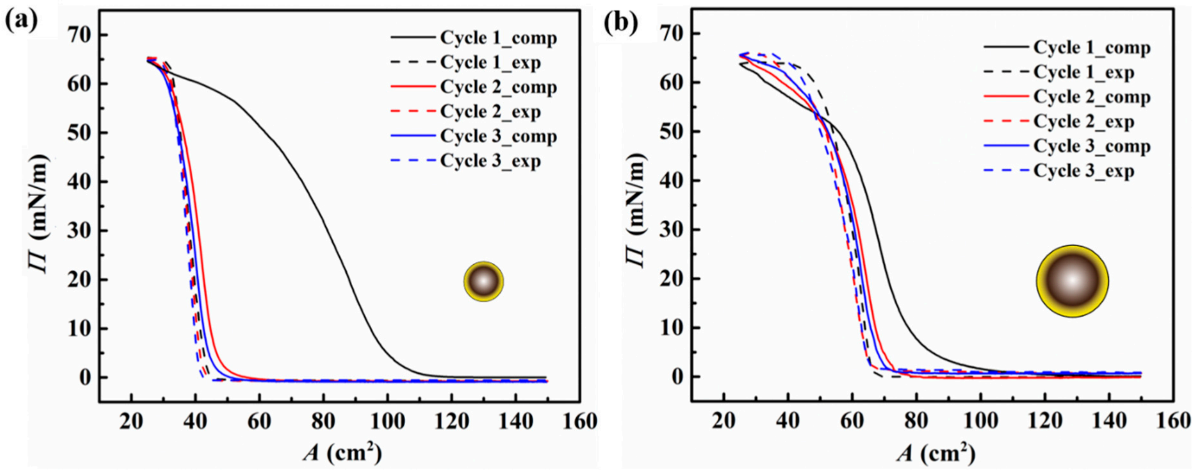

Figure S3. Plot depicting Hysteresis of interfacial particle networks examined via three successive compression-expansion cycles carried out on samples of (a) 250 nm and (b) 1000 nm particles at the air-water interface.

Shear rheology date for silica NP networks at air-water interface

Oscillatory shear measurements aid in determining the mechanical properties of the particle laden interfaces. Various tests such as strain amplitude sweep and frequency sweep are performed to characterize the interface. It is observed that, within the linear viscoelastic regime, the elastic modulus value ($G^{s\prime}$) for both the 250 nm and 1000 nm particle networks are larger than the loss modulus values ($G^{s\prime\prime}$). **Figures S4(a)** and **(b)** present the elastic values obtained during the amplitude sweep measurements for 250 nm and 1000 nm particle networks at different surface pressures (Π). It can be observed that the shear moduli values for the bigger particles are larger than those measured for the smaller particle networks. This result can be attributed to the enhanced interparticle attractions in the larger particle networks. Furthermore, the transition from the linear to non-linear regime occurs at a relatively lower strain for 250 nm compared to the 1000 nm particle network.

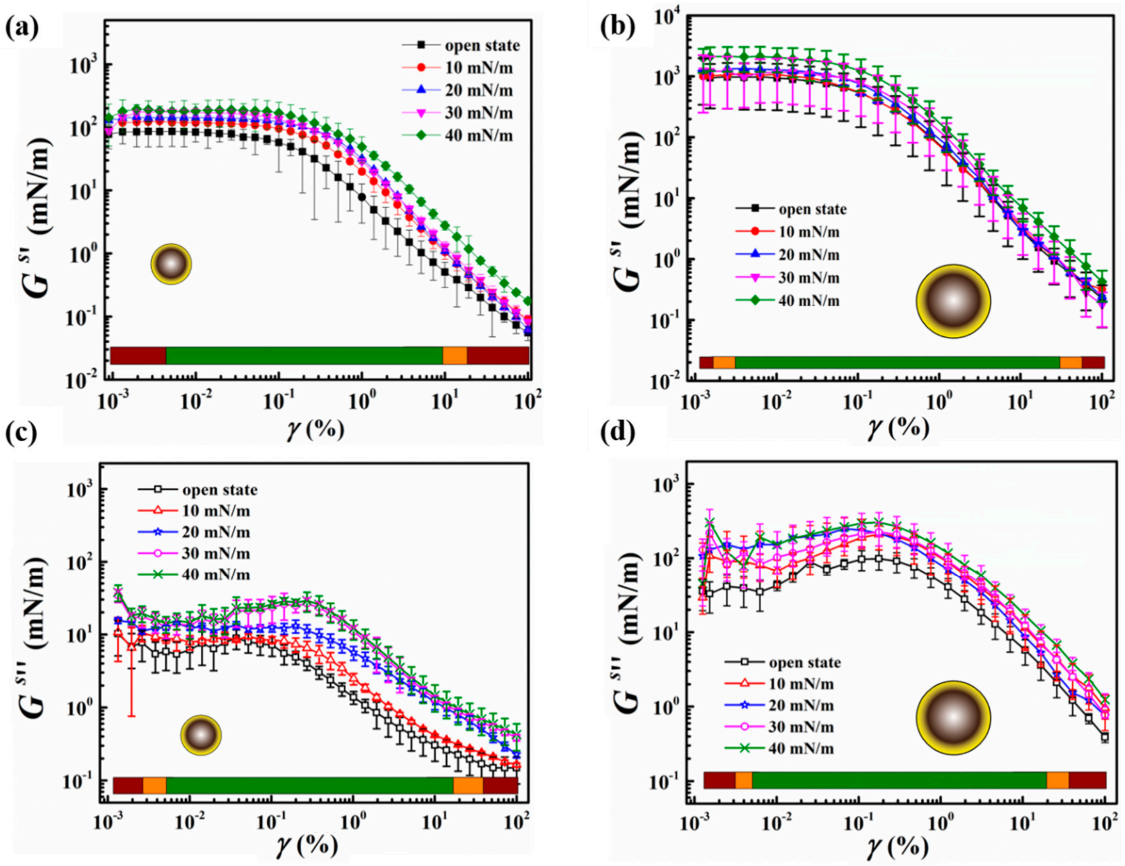

Figure S4. Complex modulus values for the different treated silica particle networks obtained during the amplitude sweep measurements. Plots (a) and (b) present the elastic modulus data and plots (c) and (d) represent the viscous modulus data for 250 nm and 1000 nm particle types, respectively.

Figure S5 (a) - (d) present the data obtained during the frequency sweep measurements. It was observed that the $G^{s\prime}$ values were larger than the $G^{s\prime\prime}$ values across both particle networks. A trend which was also observed during the amplitude sweep measurements. It can be noted that at lower frequency values there is some discrepancy in the data trend probably because of the instrument limitations. With increasing driving frequency there is a slight reduction in the $G^{s\prime\prime}$ values across the different surface pressure for both 250 nm and 1000 nm particle networks. **Figure S5 (e) - (f)** presents the complex modulus and loss tangent values for the smaller and larger particle networks at surface pressure of 30 mN/m. The loss tangent denotes the ratio of energy dissipation to energy storage (loss modulus to elastic modulus). For the smaller particle network, the loss tangent reduces as the frequency is increased and then plateaus. For the larger particle network, a similar trend is observed; with increasing external frequency, the loss tangent values reduce and

then plateau. However, it may be noted that the loss tangent value plateaus at ~10^0 rad/s for 250 nm particles, whereas for 1000 nm particles, it happens closer to ~10^1 rad/s.

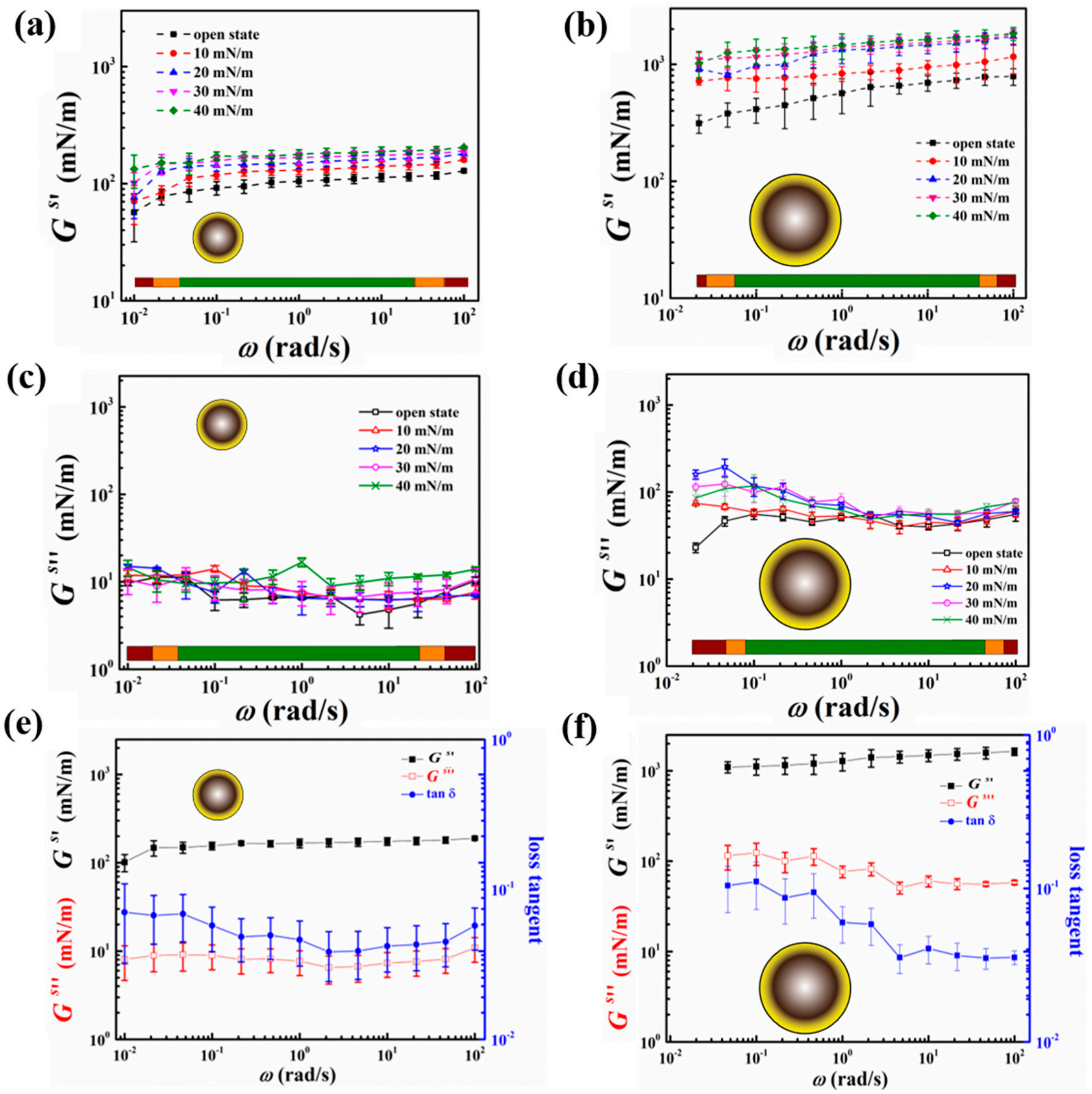

Figure S5. Rheological properties of the treated silica particle networks obtained via frequency sweep measurements. (a) and (b) display the elastic modulus of the networks present at different surface pressures, for 250 nm and 1000 nm particles, respectively; (c) and (d) illustrate the viscous modulus of the networks present at different surface pressures for 250 nm and 1000 nm particles, respectively; (e) and (f) present the elastic modulus, viscous modulus, and the loss tangent data for networks at surface pressure of 30 mN/m, and for 250 nm and 1000 nm particles, respectively.

Assumptions in the calculations of capillary interactions

Assumptions that went into the calculation of the capillary interactions as per $U_{cap} = -12\pi\gamma H^2 \cos[2(\varphi_A + \varphi_B)]\frac{r_c^4}{L^4}$ are as follows: i) the variation in the contact line undulation, H, has been taken to be ~ 1% of particle size, that is, 10 nm for 1000 nm particles and 2.5 nm for 250 nm DMDCS coated silica particles (representative values which fall on the lower range from that observed in the literatures).[6,7] Different values for two particle size have been chosen based on the direct

dependence of H as per $H \approx \frac{1}{2} R \times \Delta\theta$ for a particular particle radius, R, and contact angle hysteresis, $\Delta\theta$.[6] ii) The capillary interactions depend on the particles angle of approach towards each other, φ_A and φ_B, which have been assumed to be 0, indicating strongest attractive capillary interactions possible. iii) Here, R is the particle radius and θ is the particle contact angle at the interface, which is assumed to be 90° in these calculations based on the measurements carried out to estimate the particle wettability. Thus, for $r_c = Rsin\theta$[6], it implies $r_c \sim R$, where r_c is the radius of the three-phase contact line and R is the particle radius. Assumption that went into the calculation of the van der Waals interactions for the particles at the interface is the value of 0.5 for the fractional immersion of particles at the interface, f, as the particles in this study were taken as neutrally wetting particles.

References

(1) Lide, R. D. *Handbook of Chemistry and Physics*, 73rd ed.; CRC Press: Boca Raton, 1992.

(2) Tadmor, R.; Hernández-Zapata, E.; Chen, N.; Pincus, P.; Israelachvili, J. N. Debye Length and Double-Layer Forces in Polyelectrolyte Solutions. *Macromolecules* **2002**, *35* (6), 2380–2388.

(3) Drazin, P. G.; Reid, W. H. *Hydrodynamic Stability*; Cambridge University Press, 2004. https://doi.org/10.1017/CBO9780511616938.

(4) Matos, C.; De Castro, B.; Gameiro, P.; Lima, J. L. F. C.; Reis, S. Zeta-Potential Measurements as a Tool to Quantify the Effect of Charged Drugs on the Surface Potential of Egg Phosphatidylcholine Liposomes. *Langmuir* **2004**, *20* (2), 369–377.

(5) Renggli, D.; Alicke, A.; Ewoldt, R. H.; Vermant, J. Operating Windows for Oscillatory Interfacial Shear Rheology. *J. Rheol. (N. Y. N. Y).* **2020**, *64* (1), 141–160. https://doi.org/10.1122/1.5130620.

(6) Horozov, T. S.; Aveyard, R.; Binks, B. P.; Clint, J. H. Structure and Stability of Silica Particle Monolayers at Horizontal and Vertical Octane-Water Interfaces. *Langmuir* **2005**, *21* (16), 7405–7412.

(7) Kralchevsky, P. A.; Danov, K. D.; Petkov, P. V. Soft Electrostatic Repulsion in Particle Monolayers at Liquid Interfaces: Surface Pressure and Effect of Aggregation. *Philos. Trans. R. Soc. A Math. Phys. Eng. Sci.* **2016**, *374* (2072). https://doi.org/10.1098/RSTA.2015.0130.
